# Differential phosphorylation of LZ+/LZ- MYPT1 isoforms by PKGIα: implication for vascular reactivity

**DOI:** 10.1186/1471-2210-11-S1-P78

**Published:** 2011-08-01

**Authors:** Samantha Yuen, Ozgur Ogut, Frank V Brozovich

**Affiliations:** 1 Cardiovascular Diseases, Mayo Medical School, Rochester, MN USA

## Background

MLC phosphatase is a trimeric enzyme composed of a catalytic subunit, a 20-kDa subunit of unknown function, and a myosin targeting subunit (MYPT1). During NO stimulation, PKGIα mediated phosphorylation of MYPT1 increases MLC phosphatase activity, which produces a decrease in force. Further, alternative splicing of a 3’ exon produces two MYPT1 isoforms, which differ by the presence or absence of a leucine zipper (LZ); a LZ+ MYPT1 isoform is required for PKGIα induced smooth muscle relaxation.

## Results

To examine the influence of MYPT1 structure on the ability of PKGIα to phosphorylate the protein, we used two MYPT1 fragments, which differed only by the presence (MYPT1LZ+) or absence (MYPT1LZ-) of the LZ. Purified PKGIα phosphorylated MYPT1LZ+, but not MYPT1LZ-. Following phosphorylation, MYPT1LZ+ predominantly existed as a di-phosphorylated protein, and mass spectrometry identified S^668^ and S^695^ as PKGIα-mediated phosphorylation sites. To examine the relative rates of S^668^ vs S^695^ MYPT1 phosphorylation, these residues were mutated to either A or D. The rates of D^668^ and A^668^ MYPT1 phosphorylation were similar and slow. The D^695^ MYPT1 mutant had the highest rate of phosphorylation, while the rate of phosphorylation of the A^695^ MYPT1 mutant was intermediate between that for the D^695^ MYPT1 and either A^668^ or D^668^ MYPT1.

## Conclusion

These results suggest that PKGIα-mediated phosphorylation of S^695^ is slower than S^668^, and could suggest that PKGIα-mediated phosphorylation of S^668^ is physiologically significant for the regulation of MLC phosphatase activity. Further, MYPT1 structure has an important role in the regulation of vascular tone, and differential tissue expression of LZ+/LZ- MYPT1 isoforms contributes to the diversity in the sensitivity of smooth muscle to NO mediated vasodilatation.

